# Surface modification of a polyhedral oligomeric silsesquioxane poly(carbonate-urea) urethane (POSS-PCU) nanocomposite polymer as a stent coating for enhanced capture of endothelial progenitor cells

**DOI:** 10.1186/1559-4106-8-23

**Published:** 2013-08-23

**Authors:** Aaron Tan, Yasmin Farhatnia, Debbie Goh, Natasha G, Achala de Mel, Jing Lim, Swee-Hin Teoh, Andrey V Malkovskiy, Reema Chawla, Jayakumar Rajadas, Brian G Cousins, Michael R Hamblin, Mohammad S Alavijeh, Alexander M Seifalian

**Affiliations:** 1 Centre for Nanotechnology & Regenerative Medicine, UCL Division of Surgery & Interventional Science, University College London, London, UK; 2 UCL Medical School, University College London, London, UK; 3 Division of Bioengineering, School of Chemical & Biomedical Engineering, Nanyang Technological University, Singapore, Singapore; 4 Biomaterials & Advanced Drug Delivery Laboratory, School of Medicine, Stanford University, Stanford, CA, USA; 5 Wellman Center for Photomedicine, Massachusetts General Hospital, Boston, MA, USA; 6 Department of Dermatology, Harvard Medical School, Boston, MA, USA; 7 Harvard-MIT Division of Health Sciences & Technology, Cambridge, MA, USA; 8 Pharmidex Pharmaceutical Services Ltd, London, UK; 9 Royal Free London NHS Foundation Trust, London, UK

**Keywords:** POSS-PCU, Stent coatings, Anti-CD34 antibody, Endothelialization, Endothelial progenitor cell capture, Nanotechnology, Regenerative medicine, Biomaterials

## Abstract

An unmet need exists for the development of next-generation multifunctional nanocomposite materials for biomedical applications, particularly in the field of cardiovascular regenerative biology. Herein, we describe the preparation and characterization of a novel polyhedral oligomeric silsesquioxane poly(carbonate-urea) urethane (POSS-PCU) nanocomposite polymer with covalently attached anti-CD34 antibodies to enhance capture of circulating endothelial progenitor cells (EPC). This material may be used as a new coating for bare metal stents used after balloon angioplasty to improve re-endothelialization. Biophysical characterization techniques were used to assess POSS-PCU and its subsequent functionalization with anti-CD34 antibodies. Results indicated successful covalent attachment of anti-CD34 antibodies on the surface of POSS-PCU leading to an increased propensity for EPC capture, whilst maintaining *in vitro* biocompatibility and hemocompatibility. POSS-PCU has already been used in 3 first-in-man studies, as a bypass graft, lacrimal duct and a bioartificial trachea. We therefore postulate that its superior biocompatibility and unique biophysical properties would render it an ideal candidate for coating medical devices, with stents as a prime example. Taken together, anti-CD34 functionalized POSS-PCU could form the basis of a nano-inspired polymer platform for the next generation stent coatings.

## Background

Cardiovascular disease is the number one cause of death in the world, and it is estimated that by 2030, 23 million people will succumb annually to cardiovascular-related diseases [1]. Atherosclerosis is a subset of cardiovascular disease, and is characterized by a build up of fatty deposits in coronary arteries. Left untreated, these blocked arteries can lead to myocardial infarction (heart attack) and even death [2]. Two types of stents are currently used for treating blocked coronary arteries that have been widened by balloon dilation: bare-metal stents (BMS) and drug-eluting stents (DES). In-stent restenosis (ISR) due to intimal hyperplasia is often seen with BMS [3], while late stent thrombosis (ST) is often seen in DES [4]. ISR is often attributed to an immunological responses mounted against the metal struts, while late ST is thought to stem from drug-polymer matrix hypersensitivity and impaired re-endothelialization [5]. Efforts are currently underway to develop new and improved polymer-matrix platforms, and surface immobilized biomolecules to mitigate the risks of ISR and ST.

To this end, we have developed a proprietary nanocomposite polymer, polyhedral oligomeric silsesquioxane poly (carbonate-urea) urethane (POSS-PCU) for medical applications. POSS-PCU is a novel nanocomposite polymer, capable of functioning as a component of artificial organs [6], coatings for nanoparticles [7], and a platform on which bioactive molecules can be attached [8]. It has been used in three different first-in-man studies as a lacrimal duct [6], as a bypass graft [9], and as the world’s first synthetic trachea [10].

POSS® was initially developed by the United States Air Force as a new breed of material for aerospace engineering purposes [11, 12]. With its superior physical and mechanical properties, this composite has subsequently found its way into the biomedical sector [13, 14]. POSS-PCU consists of a PCU polymer backbone that is reacted with POSS functioning as nanofillers, augmenting its mechanical and degradative resistance properties [15]. The biocompatibility of POSS-PCU [16] and its unique biophysical properties [17] have made it an attractive candidate as a coating for cardiovascular devices [18].

**Figure 1:**
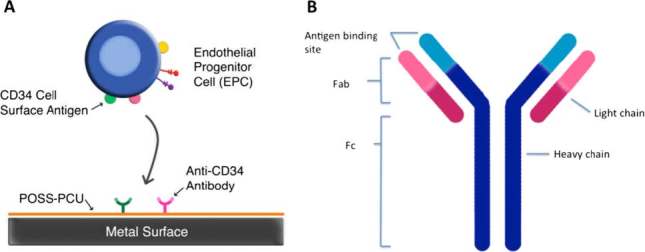
**A clinical-grade biofunctionalized polymer for coating stents. (A)** POSS-PCU nanocomposute polymer can be used to coat bare metal stents, and further functionalized with endothelial progenitor cell (EPC)-specific antibodies for enhanced endothelialization. **(B)** A schematic diagram of an anti-CD34 antibody. The Fab region binds to EPCs, while the Fc region is immobilized onto POSS-PCU.

We therefore postulated that coating BMS with the highly biocompatible nanocomposite polymer, POSS-PCU, would prevent ISR. Furthermore, functionalizing POSS-PCU with endothelial progenitor cell (EPC)-specific antibodies, (i.e. anti-CD34 antibodies), could attract circulating EPC and thereby enhance endothelialization to prevent late ST (Figure 1). Although there are a number of possible antibodies like anti-CD133 [19], and peptide motifs [20] that could attract EPCs, the main premise of selecting anti-CD34 is due to more favourable results as seen in preliminary studies done in our lab, and previous work done by other groups [21–23]. Currently, the Genous™ stent (OrbusNeich) utilizes anti-CD34 antibodies covalently attached to metal stent struts via a polysaccharide matrix coating [24], and is currently used in clinical trials. Due to the fact that POSS-PCU has been used in 3 first-in-man implants, and demonstrated favourable biological properties in terms of biocompatibility and hemocompatibility, we advocate its use as a new type of robust and non-biodegradable coating platform for stents.

This study attempted to assess the feasibility of using POSS-PCU as a polymer coating for coronary stents, as well as its potential for being an endothelial progenitor cell (EPC) capture platform, when functionalized with anti-CD34 antibodies. Fourier transform infrared spectroscopy was used to detect chemical groups in POSS-PCU films; water contact angle was used to measure surface wettability; quantum dots (QDs) tethered to secondary antibodies were used as fluorophores to detect primary antibodies on POSS-PCU, thromboelastography (TEG) was used to assess hemocompatibility; Alamar Blue was used to assess *in vitro* biocompatibility; atomic force microscopy (AFM) was used to visually characterize surface topography and quantify surface roughness; Raman spectroscopy and Raman integration maps were used to identify POSS regions, PCU regions, and antibody regions of the polymer; X-ray photoelectron spectroscopy (XPS) was used to measure and quantify surface elemental composition; EPCs were cultured onto POSS-PCU films to assess the efficacy of EPC capture; POSS-PCU-coated stents were placed in a flow circuit mimicking physiological flow conditions to assess the stability of antibody immobilization.

Therefore, the aim of this study was to use biophysical techniques to assess the surface modifications of POSS-PCU after antibody attachment, and also to assess the feasibility of using POSS-PCU-CD34 as an EPC capture platform for stent coatings.

## Methods

All reagents were purchased from Sigma Aldrich UK, unless otherwise stated. For procedures that involved the use of human blood and tissue, informed consent was obtained from healthy volunteers, and the Institutional Review Board (IRB) at the Division of Surgery & Interventional Science at University College London approved the study protocol. All experimental procedures were done in triplicates (n = 3) unless otherwise stated.

### POSS-PCU nanocomposite polymer synthesis

Synthesis of POSS-PCU for peptide functionalization has been previously described elsewhere [25]. Briefly, polyhedral oligomeric silsesquioxane (POSS®) (Hybrid Plastics Inc.) was mixed with polycarbonate polyol in a custom-built reaction flask. The mixture was heated and stirred using a mechanical stirrer. 4,4′-methylenebis (phenyl isocyanate) (MDI) and nitrogen gas were introduced into the reaction mixture to form the pre-polymer. Dimethylacetamide (DMAc) was added to the mixture. Chain extension was commenced via addition of ethylenediamine and diethylamine to yield the final product, 18% (w/w) solution of POSS-PCU. Functionalized fumed silica was then incorporated into POSS-PCU using a UIP1000-Exd Ultrasonic Mixer (Hielscher Ultrasonic GmbH).

### O-Phthalaldehyde (OPA) fluorescent amine assay

OPA assay was used to detect the presence of primary amines on POSS-PCU that would be functionalized with antibodies. 83 μl of 2-mercaptoethanol and 833 μl of borate buffer (0.05 mol/dm^3^, pH = 9) were added onto the different test samples. The mixture was transferred to a 96-well plate and left to stand for 2 hours. Thereafter, 34 μl of OPA (10 mg/ml in ethanol) was added. The plate was placed in a Fluoroskan Ascent FL microplate fluorometer/luminometer (Thermo Scientific). A 360 nm excitation filter, and a 460 nm emission filter were selected.

### Ultrasonic atomization spray system

The above-mentioned version of POSS-PCU has a high viscosity, and must be diluted for the purposes of using it in the ultrasonic spray atomization system. Briefly, 22 g of tetrahydrofuran (THF) was added to 2 g of POSS-PCU.

The parameters used for the polymer coating were written into the program of the MediCoat DES 1000 Ultrasonic Spray System (Sono-Tek Corporation USA). Polymer solutions were fed into the nozzle, through a syringe, of the ultrasonic atomization spray system. Nitrogen gas (BOC Industrial Gases) pressure was set at 4.5 PSI. Ultrasonic power was set to 0.24 W. Rate of syringe injection was set at 0.1 ml/min. Translational movement of mandrel was set at 2.5 mm/sec, and rotational movement was set at 115 rpm.

Platinum chromium intra-arterial stents (Boston Scientific USA) with diameters of 3.5 mm and lengths of 20 mm were placed on mandrels, in such a way that half of the stent length “overhangs” out from the mandrel, enabling it to be spray coated in both the luminal and abluminal area while the mandrel rotates. Drying gas was utilized during this procedure at 1.0 PSI. The half-coated stent was then placed in a drying oven (Binder GmbH) at 65°C for 3 hours to allow for solvent evaporation. The coating process was repeated for the other half of the stent.

Polymer films (for cell culture) were also fabricated a similar manner, and the rotating mandrel was spray coated with the polymer using identical parameters mentioned above. A vertical incision was made lengthwise on the coated mandrel, and the polymer films were carefully peeled off.

### Covalent bonding of anti-CD34 antibodies onto POSS-PCU

The protocol for covalent bonding of peptide motifs on POSS-PCU was previously developed by de Mel in our lab, and in-depth discussion can be found elsewhere [25]. Briefly, 0.008 g of *N*-(3-Dimethylaminopropyl)-*N*’-ethylcarbodiimide hydrochloride (EDC), 0.0115 g of *N*-hydroxysuccinimide (NHS), and 0.05 g of succinic acid were placed in a 50 ml conical tube. To this, 20 ml of phosphate-buffered solution (PBS) was added. The mixture was placed on a roller mixer (Stuart Equipment) for 3 hours, to allow activation.

Circular-cut discs of polymer sheets were placed in a 24-well plate. 500 μl of the above-mentioned EDC-NHS-PBS mixture was pipetted onto each polymer sheet in the wells. The well plate was wrapped in aluminium foil to avoid light, and placed on a Luckham R100 Rotatest Shaker (Richmond Scientific Ltd.) for 3 hours.

500 μl of PBS, and 5 μl of mouse anti-CD34 concentrate (2 μg/ml) (Life Technologies) were pipetted into an eppendorf tube. Equal amounts of the antibody solution were pipetted into 24-well plates. The well plate was wrapped in aluminium foil and placed on a shaker for 30 minutes. It was then transferred to a 4°C fridge and left for 24 hours. After 24 hours, the polymer discs were washed with PBS.

POSS-PCU-coated stents were also covalently-bonded to anti-CD34 antibodies, using a similar protocol as mentioned above.

### Fourier transform infrared (FTIR) spectroscopy

Chemical groups were detected using ATR-FTIR with a Jasco FT/IR 4200 spectrometer (JASCO Analytical Instruments). Parameters were set at 20 scans at a 4 cm^-1^ resolution, with a wavenumber range of 600 cm^-1^ to 4000 cm^-1^.

### Scanning probe AFM confocal raman spectroscopy

Raman and AFM scanning were performed using NT-MDT NTEGRA Spectra® system with an upright Raman microscope and a universal head. AFM scanning was done in semi-contact mode with commercial rounded cantilevers for large scans, purchased from MicroMasch (R ~ 40 nm, k = 5.7 N/m). Raman scanning was done in backscattering geometry with a Mitutoyo long-working distance objective (100×, 0.7 NA). The excitation source was a 473 nm solid-state Cobolt Blues® laser with power at the sample being 2 mW. Acquisition time per step was 10s and step size was 0.5 microns. Optical images of the area were captured using the same objective.

### Water contact angle

A KRÜSS DSA 100 (KRÜSS GmbH) system was used for static water contact angle measurement, using a sessile drop method. Sterile deionized water was used as a solvent, with droplet volume of 3 μl. A highly hydrophobic material, Teflon® (DuPont USA) and a highly hydrophilic material, Acuvue® (Johnson & Johnson) served as controls.

### Thromboelastography (TEG)

The effect of polymer material on blood coagulation kinetics was assessed using a TEG® 5000 Thromboelastograph® Hemostasis Analyzer System (Haemonetics Corporation USA). Cuvettes were spray coated with POSS-PCU and further conjugated with anti-CD34 antibodies in the protocol mentioned above. Uncoated cuvettes served as controls. Whole blood was obtained from healthy volunteers and 320 μl of whole blood were pipetted into each cuvette.

### Scanning electron microscopy (SEM)

Samples were mounted on an aluminium stub and sputter-coated with gold via vapour deposition using SC500 (EM Scope), and imaged using a Philips 501 SEM (Cambridge, UK).

### X-ray photoelectron spectroscopy (XPS)

The analysis of the samples was carried out using a Thermo Scientific Theta Probe XPS recently calibrated in November 2012. Monochromatic Al Kα X-ray (hν = 1486.6 eV) was employed with an incident angle of 30° with respect to surface normal. Photoelectrons were collected at a take-off angle of 50° with respect to surface normal. The analysis area was approximately 400 μm in diameter while the maximum analysis depth lies in the range of 4 - 10 nm. Survey spectra and high-resolution spectra were acquired for surface elemental identification and for chemical state identification, respectively. For chemical state analysis, a spectral deconvolution was performed by a curve-fitting procedure based on a Lorentzian function, and broadened by a Gaussian function.

### Endothelial progenitor cell (EPC) and human umbilical vein endothelial cell (HUVEC) culture

Two cell lines were selected for this study: endothelial progenitor cells (EPCs) and human umbilical vein endothelial cells (HUVECs). EPCs were selected as we wanted to find out the capturing efficacy of CD34-POSS-PCU, while HUVECs were used to confirm that CD34-POSS-PCU were able to support the growth and proliferation of endothelial cells. Both cell lines were cultured in the same type of cell culture medium to ensure experimental consistency.

Details of EPC extraction were reported previously by our group [25, 26]. Whole blood was obtained from healthy volunteers after signing an informed consent document. Approximately 20 ml of whole human blood was placed in eight 2.7 ml light blue-capped Vacutainer® citrate tubes (BD USA). 3 ml of Histopaque was added to six 15 ml centrifuge tubes, and 3 ml of whole blood was layered onto the Histopaque. The material at the opaque interface was transferred into 2 clean centrifuge tubes. 10 ml of HBSS (Life Technologies) was added, mixed gently and centrifuged at 250 *g* for 10 minutes. The supernatant was discarded and the pellet re-suspended in 5 ml HBSS and mixed gently. This centrifugation and supernatant-discarding process was repeated 2 more times. The cells were then re-suspended in 5 ml cell culture medium M199 (Life Technologies) with 10% FBS (Life Technologies), and 1% penicillin and streptavidin (Life Technologies). Cells were counted using Trypan blue exclusion dye, using 10 μl Trypan blue, and 10 μl cell suspension. Cells were seeded at a density of 1.0 × 10^6^ cells per well in a 24-well plate. It was then incubated at 37°C with 5% CO_2_ for 21 days. Culture medium was replenished every 3 days, and cells were examined under light microscopy every 3 days.

HUVEC extraction was described extensively by our group in a previous report [27, 28]. The protocol was modified to fit our experimental needs in this study. Briefly, human umbilical cords were obtained immediately upon delivery of newborn infants, from the Department of Obstetrics and Gynaecology at the Royal Free London NHS Foundation Trust Hospital, after patients had signed an informed consent document, and approved by the IRB at UCL. Similar to the above-mentioned protocol for EPCs, cells were seeded at a density of 1.0 × 10^6^ cells per well in a 24-well plate. It was then incubated at 37°C with 5% CO_2_ for 21 days. Culture medium was replenished every 3 days, and cells were visualized every 3 days.

### AlamarBlue® assay

AlamarBlue® (Life Technologies) was added to the cell culture medium at a volume to volume concentration of 10%. After 7 days, culture plates were washed with PBS and 1 ml was added to each well. 100 μl was aspirated after 4 hours. Fluorescence was measured using a Fluoroskan Ascent FL microplate fluorometer/luminometer (Thermo Scientific). A 360 nm excitation filter, and a 460 nm emission filter were selected.

### Dynamic flow at physiological conditions

A home-built flow circuit was used to mimic physiological flow conditions in the human body. An electromagnetic pump and a variable height fluid reservoir maintained pressure at around 120 mmHg / 80 mmHg, and “heart beat” at 84 beats per minute. An ultrasound Doppler probe was used to monitor and maintain flow conditions using a Light Patient Monitor (Datex Engstrom). Culture medium with peripheral blood cells (1× 10^6^) were used in the flow circuit. Viscosity was approximated to that of blood, by adding 8% low molecular weight Dextran (77000 Da). Anti-CD34 POSS-PCU coated-stents, POSS-PCU-coated stents, POSS-PCU films, anti-CD34 POSS-PCU films, and IgG-POSS-PCU films were placed in a microtubule and subject to physiological flow conditions for 28 days.

### Quantum dots for confocal imaging

Quantum dots (QDs) were manufactured in-house by our lab in a study described previously [29], and modified to suit our experimental needs for this study. Briefly, 200 μl of QD (1 mg/ml) was mixed with 200 μl EDC (1 mg/ml) and 200 μl NHS (1 mg/ml) in PBS for 30 minutes at room temperature. 100 μL of anti-*x* solution (where *x* denotes the type of antibody used) was added to the mixture and mixed for 1 hour at room temperature. To separate the reagent and unconjugated CdCoTe/MSA/ QDs, 100 kDa Amicon® Ultra-centrifugal filters (Millipore Corporation) with UV monitoring at 280 nm of the retained samples was used. The purified samples were collected and stored at 4°C until further use. The sample was further characterized by NIR fluorescence and FTIR spectroscopy.

Cells were fixed with 2% formaldehyde (PFA) using the following method. Briefly, cells were washed twice with 0.1% PBS-Tween 20 at room temperature. They were then treated with 2% PFA for 20 minutes, and washed 3 times with 0.1% PBS-Tween 20 for 5 minutes per wash. Cells were permeabilized with 0.5% PBS-Tween 20 for 15 minutes. They were then further washed 3 times with 0.1% PBS-Tween 20 for 5 minutes per wash. Cells were blocked and incubated with quantum dots using the following methods. PBS-Tween 20 was removed. Cells were blocked with 500 μl of 1% BSA (in PBS-Tween 20) for 20 minutes at 4°C, and then washed with PBS-Tween 20. 500 μl of 0.25 μg/ml of red QD-Ab (VEGFR_2_) and green QD –Ab (CD34) to each well and incubated for 2 hours in the dark at 4°C. Cells were then visualized and counted. Samples were washed with PBS-Tween 20 to remove unwanted QDs. 300 μl of diluted DAPI was added to each well and incubated for 5 minutes. They were then washed with PBS-Tween 20.

Images were acquired by a fluorescent microscopy unit (Nikon Eclipse TE 300). The PCM scanning head was mounted on an inverted optical microscope (Nikon Eclipse TE 300), which can operate in fluorescence, reflection and phase contrast modes, and it was fitted with a Plan Fluor dry objective (20×/NA = 0.5). Lasers at 488 nm and 543 nm were the sources, housed in a common module, providing the excitation beams that were delivered to the scanning head through a single-mode optical fiber. Photomultiplier (PMT) tubes were placed within the control unit, and the collected light arrived via high-transmission optical fibers.

### Curve fitting and statistical analyses

Curve fitting (least squares method) and statistical analyses were conducted at 95% confidence interval using MATLAB® (MathWorks Inc.). Statistical significance testing was conducted using unpaired Student’s *t*-test. *p*-values of less than 0.05 were considered statistically significant.

## Results and discussion

### Detection of amine on antibody-functionalized POSS-PCU via OPA assay

**Figure 2:**
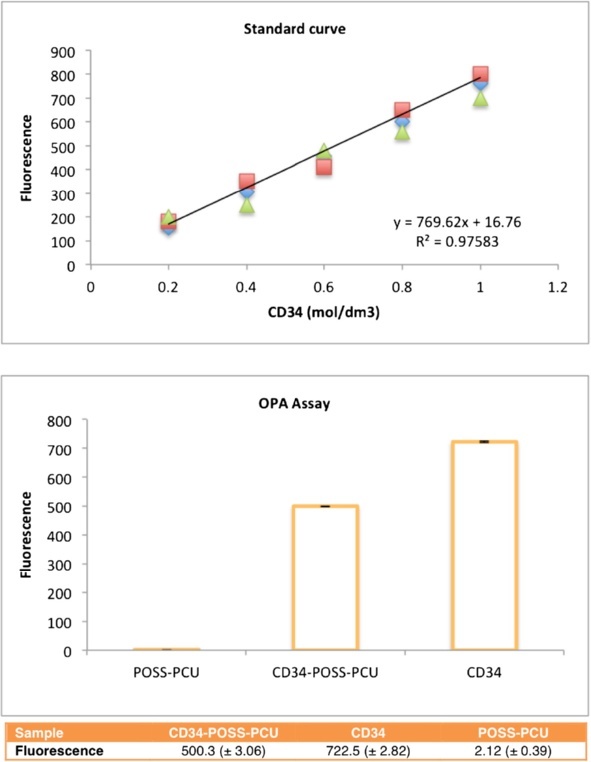
**Detection of amine groups on anti-CD34 antibody.** OPA assay showed the presence of amine groups on POSS-PCU. This value was somewhat less that pure anti-CD34, indicating a certain about of loss during functionalization.

The OPA assay revealed a higher fluorescence signals from POSS-PCU-CD34 compared to POSS-PCU (Figure 2). This indicated that amine groups were detected on the surfaces of POSS-PCU-CD34. However, it could be seen that fluorescence of POSS-PCU-CD34 was lower than pure CD34, indicating a certain level of loss during functionalization. However, preliminary analyses indicated that functionalization efficiency was consistent at 70%, indicating a sufficiently high level of antibody functionalization. As CD34 alone displayed the highest fluorescence levels, a standard curve was plotted using known concentrations of anti-CD34 in order to ascertain the concentration of CD34 grafted onto POSS-PCU.

### FTIR spectroscopy

**Figure 3:**
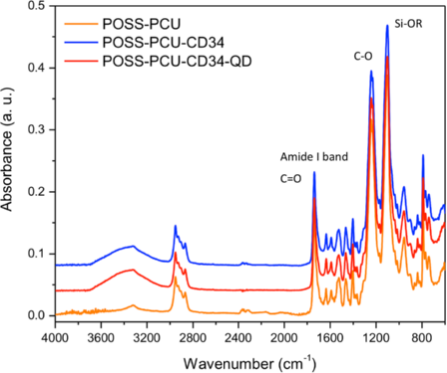
**Detecting chemical groups via FTIR.** FTIR spectra revealed that incorporation of NH2-FS did not alter the spectral read-outs. Amide I band was detected in anti-CD34 antibodies.

The peak at 1100 cm^-1^ represents the Si-OR functional group in the POSS molecule, as POSS has a chemical formula of (RSiO_1.5_)_n_ (Figure 3). The peak at 1700 cm^-1^ represents the carbonyl group, C = O, in the urea hard segment and polycarbonate soft segment of the POSS-PCU molecule. The peak at 1200 cm^-1^ represents the C-O group in urea hard segment and polycarbonate soft segment of the POSS-PCU molecule. FTIR studies were consistent with previous studies on POSS-PCU [30], which showed that these surface modifications did not alter the chemical integrity of POSS-PCU. However, due to the fact that the antibodies attached on the surface would only be bioactive under physiological solutions (aqueous solutions), we used Raman spectroscopy to give complementary information, because in FTIR, water absorbs strongly in the IR spectrum.

### Assessment of surface topography modifications using AFM and SEM

**Figure 4:**
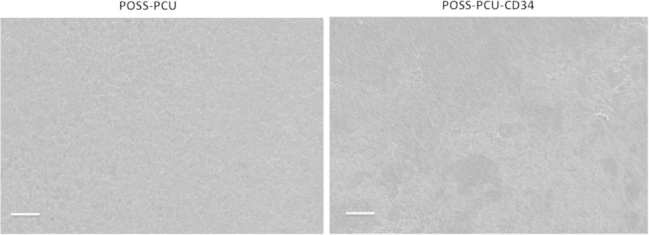
**SEM images of POSS-PCU.** Pure POSS-PCU films displayed a flake-like surface. Immobilization with anti-CD34 antibodies causes the surface to adopt a more ridge-like appearance, possibly due to protein aggregations. Scale bar represents 20 μm.

**Figure 5:**
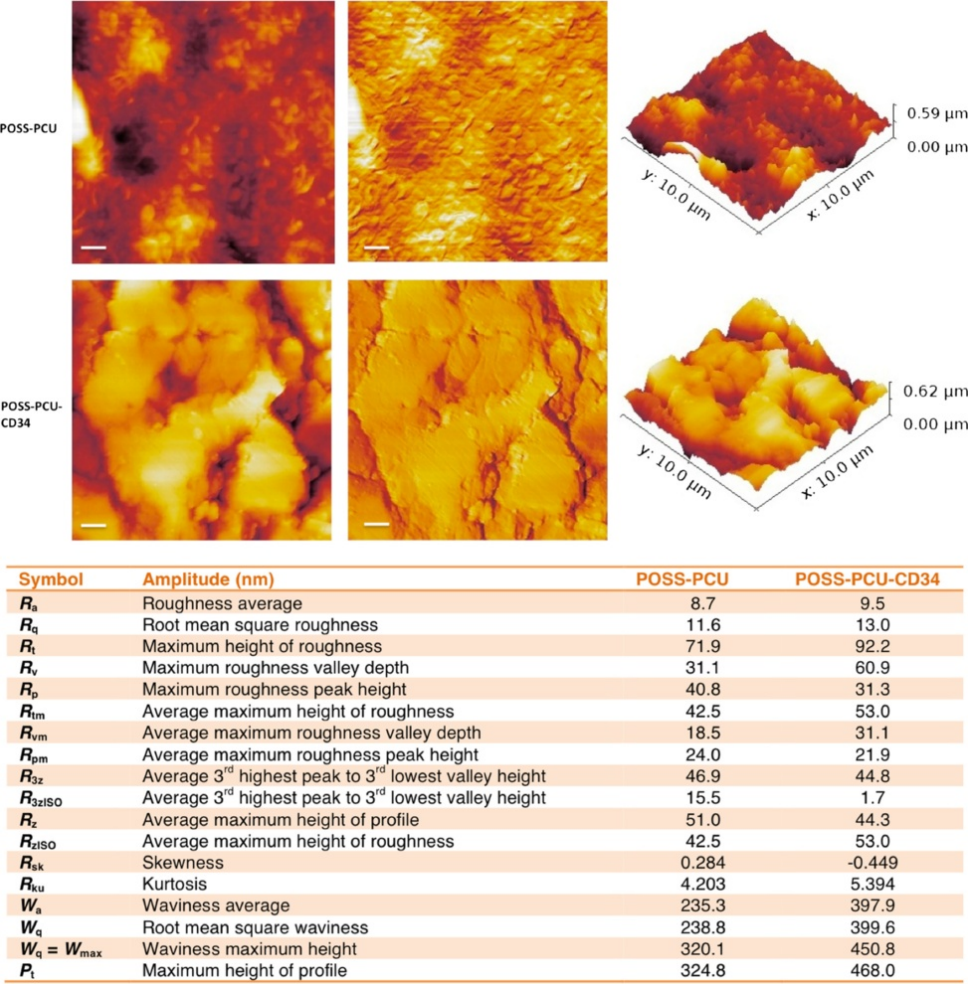
**AFM images of POSS-PCU.** Pure POSS-PCU films displayed a topography with “spikes”. Anti-CD34 antibody immobilization changes the topography to a more ridge-like appearance. This is largely consistent with SEM images. Scale bar represents 1 μm.

Changes in surface topography were observed after antibody conjugation. Surfaces adopted a granite-like cobblestone appearance after antibody conjugation, possibly due to protein aggregates (Figure 4). The tertiary structures of antibodies can be observed to modify the surfaces of POSS-PCU films, converting the bulbous-like structures on POSS-PCU into ridges (Figure 5). The surface area to volume ratio was also seen to decrease as the “micro-pillars” seen on pure POSS-PCU surfaces became larger and less numerous. SEM images revealed a flake-like pattern on POSS-PCU, while POSS-PCU-CD34 had a ridge-like appearance. In agreement with our previous studies, the bulbous structures on surface of POSS-PCU were POSS molecules that had migrated to the top of the surface during solvent evaporation [31].

### Confocal AFM-raman spectroscopy

**Figure 6:**
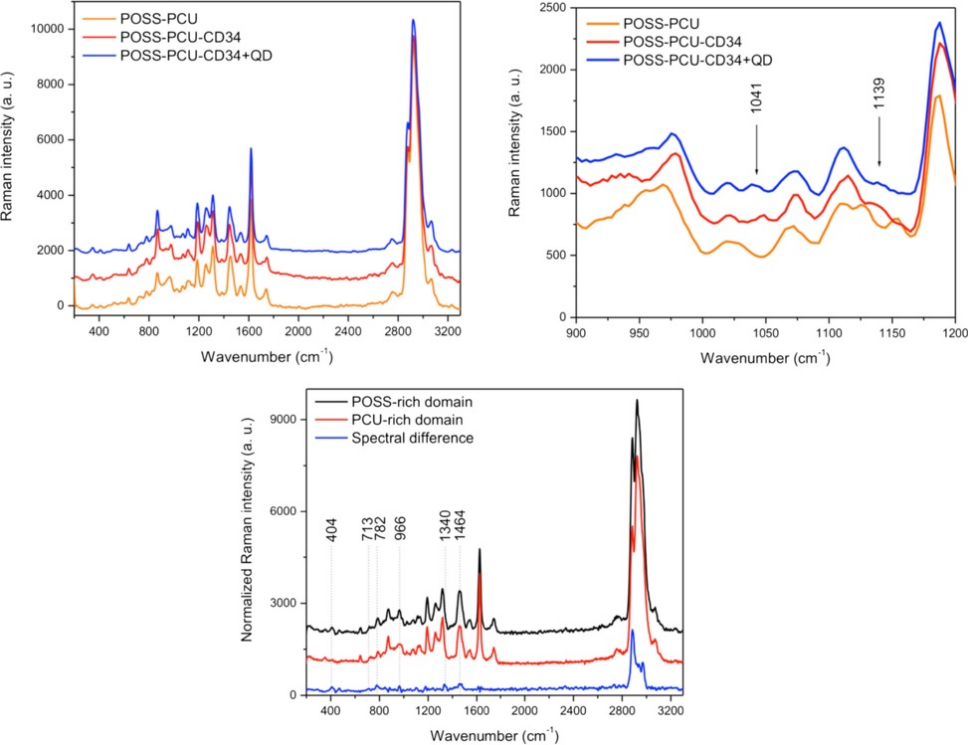
**Raman spectroscopy.** Raman intensity at the POSS regions were especially strong. Similar Raman shifts were seen in both POSS-PCU and POSS-PCU-CD34 samples due to the strong POSS signatures in the polymer. The spectral difference between POSS and PCU were used to create Raman integration maps.

All the Raman spectra generally had very similar intensities, however they were normalized to the intensity of their C-C peak at 1619 cm^-1^, which comes primarily from the PCU part of the material, and thus should not change between the different compositions. The spectra were offset vertically for easier viewing. The positions of the characteristic peaks were calibrated by crystalline Si peak and were not seen to drift during the measurement (Figure 6). The assignments were done using established methods [32–34] and references therein (Table 1).

**Table 1:** Raman and IR frequencies of POSS-PCU nanocomposite

Peak assignment	ν(FTIR), cm^-1^	ν(Raman), cm^-1^
Si-O-H bending	-	404 (m)
p-substituted C-H deformation of aromatic ring	790	777 (m)
		867 (s)
p-substituted C-H bending of aromatic ring	1018	1022 (vw)
	958	975 (m)
Urethane C-O stretching	1065	1067 (w)
Cage Si-O-Si stretching	1111	966 (w)
Carbonate C-O-C stretching	1242	1253 (s)
CHN deformation	1529	1533 (m)
C-C stretching	1402	1620 (s)
p-substituted stretching of aromatic ring	1635	1648 (sh)
	1591	1591 (sh)
	1462	1451 (s)
Carbonate C = O stretching from carbonate	1738	1738 (m)
C-H symmetric stretching	2802	2751 (m)
C-H asymmetric	2937	2967 (sh)
stretching		2933 (sh)
(metha)		2919 (s)
(ortho)		2880 (s)
N-H asymmetric stretching	3323	3327 (vw)

Legend: *vw* very weak, *w* weak, *m* medium, *s* strong, *sh* shoulder.

The Si-O vibrations are clearly discernible from the spectrum, which results after the subtraction of the PCU-rich domain spectrum from a POSS-rich one (Figure 6C). Most prominent peaks have been marked in the figure. The Si-O cage vibration has been assigned to the peak at 996 cm^-1^, while Si-O-H bending vibration has been assigned to 404 cm^-1^. The amine functionality of the silica particles is too weak to be distinguished from the control material, since it is located right next to the very strong C-C band at 1619 cm^-1^. It is important to note here that no peak can be observed in the vicinity of 2050 cm^-1^, which would correspond to Si-H vibration. This means that all of the silsesquioxane has been bound to PCU polymer. Other bands could be assigned as antisymmetric (713 and 782 cm^-1^) and symmetric (1340 and 1464 cm^-1^) Si-C-H bending vibrations.

The POSS-PCU-CD34 and POSS-PCU-CD34-QD samples did not show any significant differences in their Raman spectra, however the former appeared to degrade faster under laser beam than the control sample (POSS-PCU). This was not observed (or at least to a much lesser extent) with the latter sample (CD34-QD). There was a minimal gain, if any, of the intensity of those bands in the QD sample.

**Figure 7:**
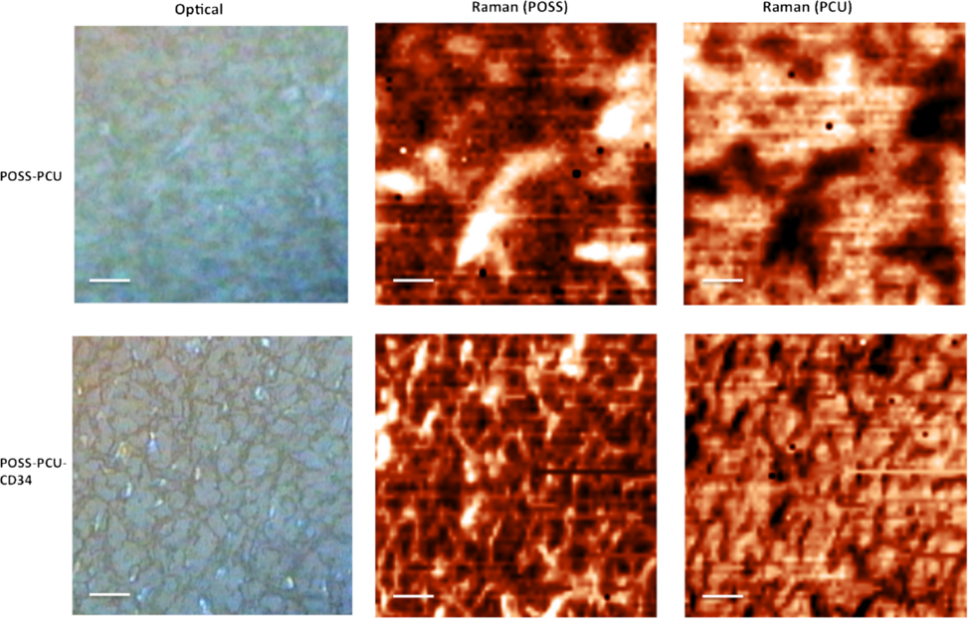
**Comparison of Raman integration maps.** Optical images and Raman maps revealed a modified surface after anti-CD34 antibody immobilization. POSS-PCU-CD34 had a granite-like appearance on both optical and Raman integration. Detection of POSS and PCU –rich regions also revealed a chemically heterogeneous surface. Scale bar represents 5 μm.

**Figure 8:**
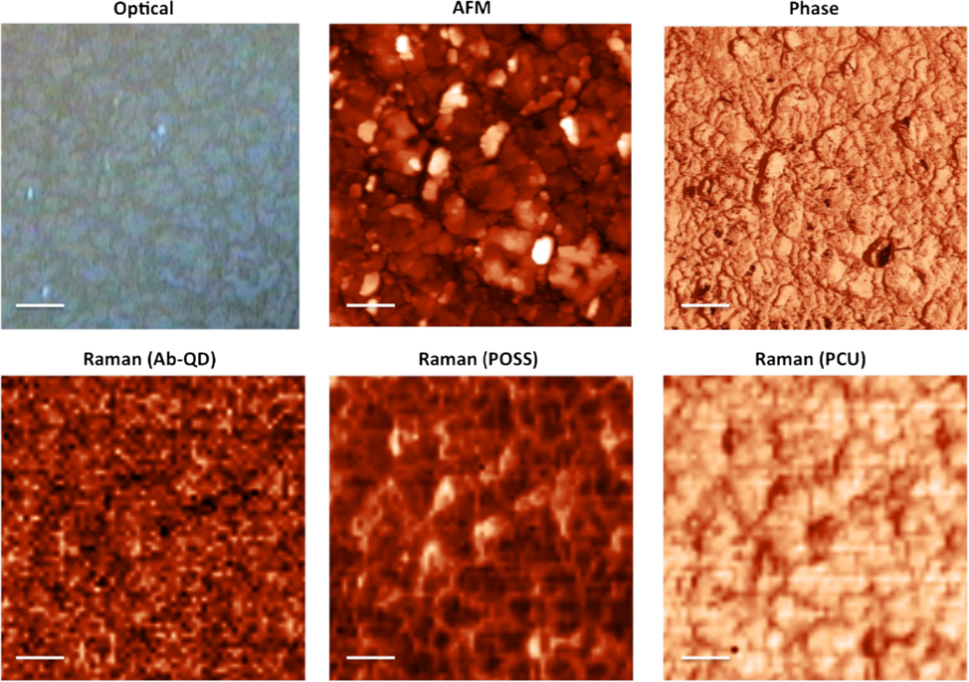
**Raman integration maps of POSS-PCU-CD34.** Raman AFM shows a cobblestone-like appearance, with phase AFM revealing a textured-surface topography. Antibody-quantum dot regions were tracked using Raman, with integrated maps showing it to be highly dispersed. Scale bar represents 5 μm.

Raman integration maps were also constructed to assess the POSS-rich and PCU-rich regions (Figure 7). After antibody attachment, optical images showed that the polymer displayed a cobblestone-like pattern (Figure 8). This is in agreement with SEM and 3D AFM images which showed a ridge-like pattern. Both the POSS-rich and PCU-rich regions can also be seen to be more dispersed after antibody attachment. More in-depth Raman integrated studies were done on POSS-PCU-CD34 with QD attached to its surface. We hoped that tethered QDs, which we used for fluorescent detection of antibodies would amplify the Raman signals at 1041 and 1139 cm^-1^ and result in an integration map with higher contrast. Raman AFM images also showed a cobblestone-like appearance, in agreement with optical images. Phase AFM images also showed the stiffness variability on the surface, and had a similar pattern to Raman AFM and optical scans. Antibody-QDs were also mapped using Raman according to their characteristic peaks (Figure 6B), and it could be seen that the antibodies were well-dispersed throughout the surface of the polymer.

### Detection of antibody engraftment via XPS

**Figure 9:**
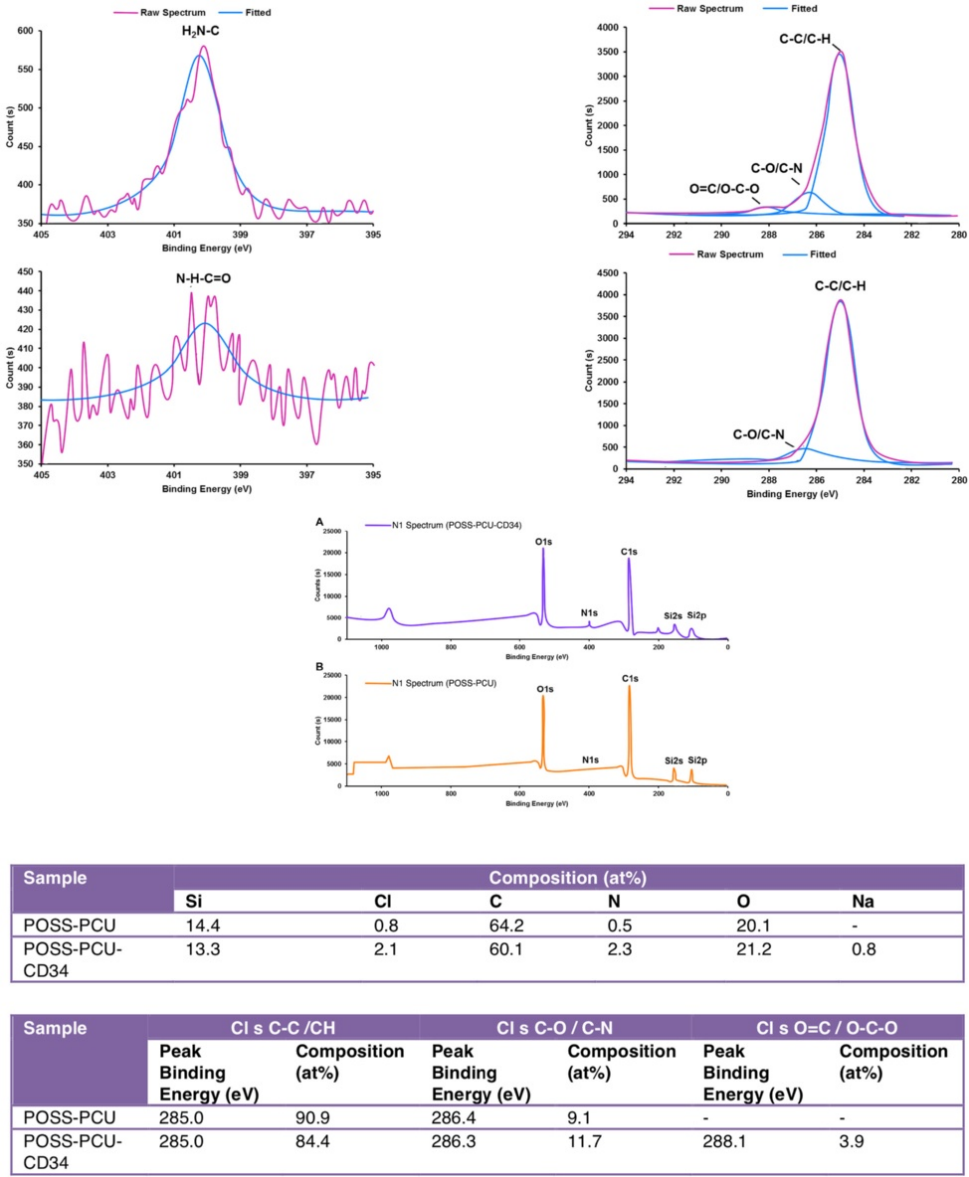
**Detection of antibody engraftment via XPS.** Atomic composition of POSS-PCU-CD34 showed a higher percentage of N compared to POSS-PCU, indicating presence of antibodies on the surface.

C, O, Si, Cl and N were detected on the surface of POSS-PCU sample (Figure 9). Part of the O & Si atoms were from POSS, while C, part of O and N were from PCU. N is likely to be present in H-N-C = O environment based on the binding energy (BE) of N1s.

C, O, Si, N, Cl and Na were detected on the surface of POSS-PCU-CD34 sample. Part of O & Si are from POSS, while C, part of O and a small part of N were from PCU. A large portion of N was from the antibody and was present in H2-N-C environment. The higher N% detected in POSS-PCU-CD34 (2.3%) than that in POSS-PCU (0.5%) suggests successful grafting of antibody on the surface of POSS-PCU. Na and Cl were likely to be present as NaCl, which were present in the anti-CD34 concentrate. Literature has shown that XPS is a viable technique for detecting the attachment of antibodies on surfaces [35].

### Surface wettability

**Figure 10:**
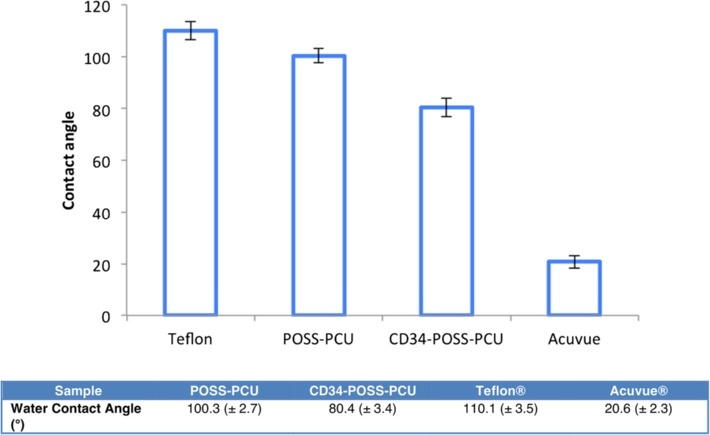
**Reduction of water contact angle.** Anti-CD34 antibody immobilization on the surface of POSS-PCU renders the surface less hydrophobic, compared to POSS-PCU. This is due to the high energy polar groups of proteins present in antibodies.

Water contact angle measures the hydrophobicity/hydrophilicity of a material. POSS-PCU displayed a contact angle of 100.3° (± 2.7) suggesting a certain degree of hydrophobicity (Figure 10). After conjugation with anti-CD34 antibodies, POSS-PCU displayed a reduced hydrophobicity, shown by a reduction of contact angle to 80.4° (± 3.4). A highly hydrophobic material, Teflon® (DuPont, UK)was used as a positive control, and displayed a water contact angle of 110.1° (± 3.5). A highly hydrophilic material, Acuvue® (Johnson & Johnson Medical Ltd, UK) was used as a negative control, and displayed a water contact angle of 20.6° (± 2.3). Tests were statistically significant from controls (p < 0.05). Hence, the water contact angle results indicated that surfaces of POSS-PCU became less hydrophobic after antibodies were attached on the surface. This is due to the polar groups on antibodies, conferring a higher surface energy, thereby lowering the water contact angle. Previous studies also showed a decrease in water contact angle after antibody attachment onto polymer surfaces [21]. However, it is important to note that the effect of water contact angle on biocompatibility / hemocompatibility is still open to debate. Therefore, rather than assigning the label of a material being biocompatible / hemocompatible simply by measuring its water contact angle, we have shown that the surface modifications via antibody attachment resulted in a reduction in water contact angle.

### Assessment of hemocompatibility

**Figure 11:**
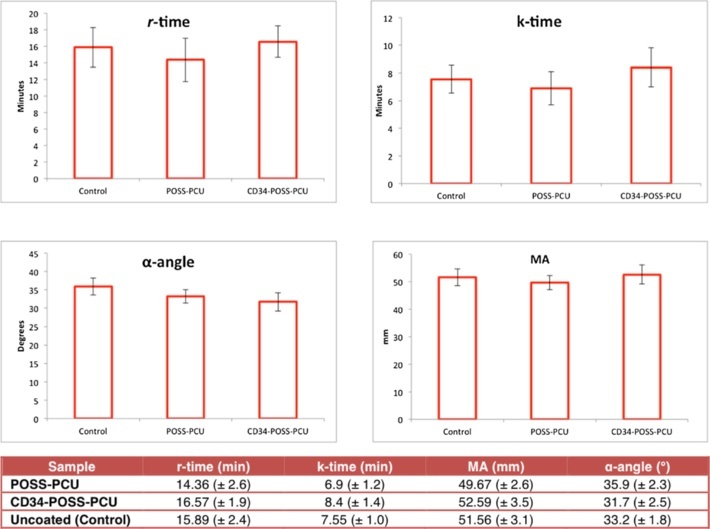
**Assessment of hemocompatibility via TEG.** TEG revealed that cuvettes coated with POSS-PCU and CD34-POSS-PCU did not significantly deviate from uncoated cuvettes. This indicates that polymer coatings did not acutely affect blood coagulation kinetics.

The TEG results revealed that POSS-PCU-coated TEG cups did not deviate significantly from control, suggesting that POSS-PCU did not adversely affect clotting kinetics of human blood (Figure 11). CD34-POSS-PCU were also coated on TEG cups, and their *k*-values, MA values, *r*-values and α-angles were not statistically significant from uncoated TEG cups, indicating hemocompatibility in terms of blood coagulation kinetics. *r*-time is the reaction time, and it represents the time until the first sign of clot is detected. Uncoated TEG cups had an *r*-time of 15.89 (± 2.4) minutes; POSS-PCU coated cups had an *r*-time of 14.36 (± 2.6) minutes; CD34-POSS-PCU coated cups had an *r*-time of 16.57 (± 1.9) minutes. Tests were not statistically significant from control (*p* > 0.05). *k*-time measures the speed at which the clot forms a size of 20 mm. Uncoated TEG cups had a *k*-time of 7.55 (± 1.0) minutes; POSS-PCU coated cups had a *k*-time of 6.9 (± 1.2) minutes; CD34-POSS-PCU had a *k*-time of 8.4 (± 1.4) minutes. Tests were not statistically significant from control (*p* > 0.05). Maximum amplitude (MA) is the width of the curve at the widest point, and gives information about clot strength. Uncoated TEG cups had an MA of 51.56 (± 3.1) mm; POSS-PCU coated cups had an MA of 49.67 (± 2.6) mm; CD34-POSS-PCU had an MA of 52.59 (± 3.5) mm. Tests were not statistically significant from control (*p* > 0.05). α-angle measures the rate of increase of elastic shear modulus. Uncoated TEG cups had an α-angle of 35.9° (± 2.3); POSS-PCU coated cups had an α-angle of 33.2° (± 1.8); CD34-POSS-PCU coated cups had an α-angle of 31.7° (±2.5). Tests were not statistically significant from control (*p* > 0.05). These results collectively indicated that, as POSS-PCU and CD34-POSS-PCU coated cups did not deviate significantly from uncoated TEG cups, therefore they had a negligible effect on coagulation kinetics, indicating high a degree of hemocompatibility.

TEG measures the coagulation kinetics of blood in real-time, and is used in cardiac and transplant surgery to assess if patients have a clotting disorder. Therefore, we wanted to assess the suitability of POSS-PCU as a blood-contacting interface, testing it against the industry-standard TEG cuvettes. Results of POSS-PCU and CD34-POSS-PCU did not deviate significantly from uncoated cups, indicating that they did not adversely interfere with normal clotting kinetics.

### Endothelial progenitor cell capture

**Figure 12:**
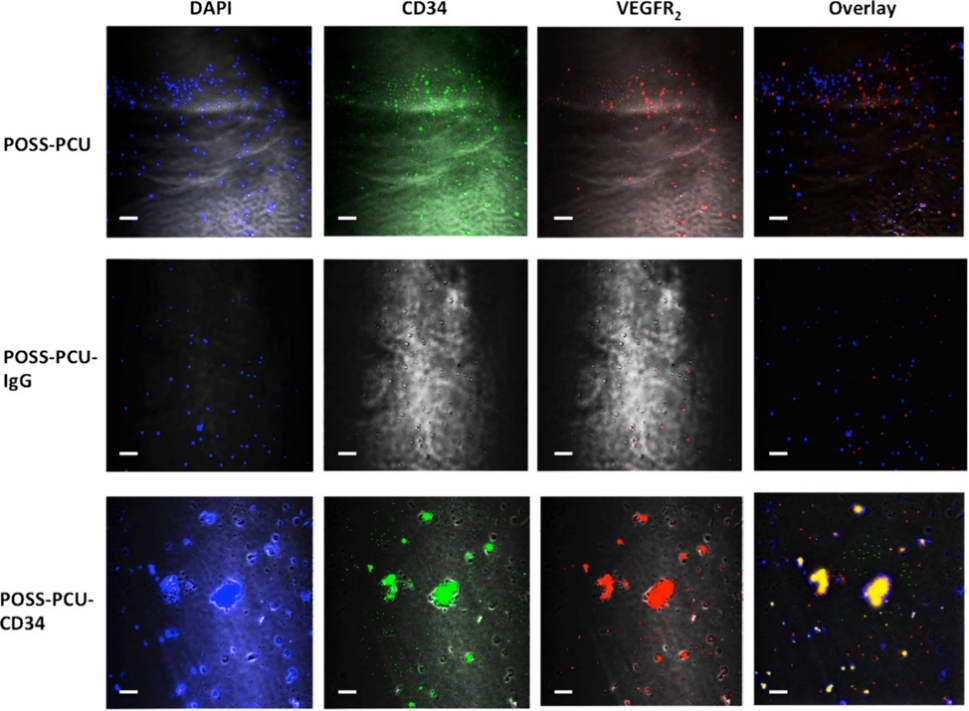
**EPC staining with anti-CD34 and VEGFR**_**2**_**.** Scale bar represents 40 μm. Compared to POSS-PCU and POSS-PCU-IgG, POSS-PCU-CD34 displayed a higher density of cell adherence which were positive for CD34 and VEGFR_2_.

**Figure 13:**
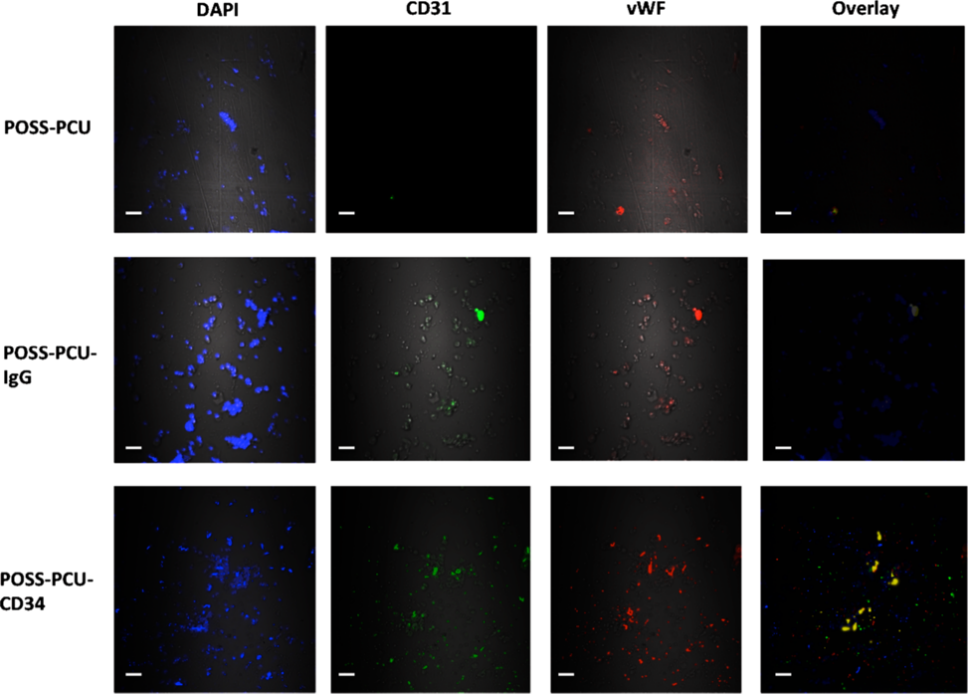
**EPC staining with CD31 and vWF.** Compared to POSS-PCU and POSS-PCU-IgG, POSS-PCU-CD34 displayed a higher degree of adherent cells that were positive for CD31 and VWF. Scale bar represents 40 μm.

**Figure 14:**
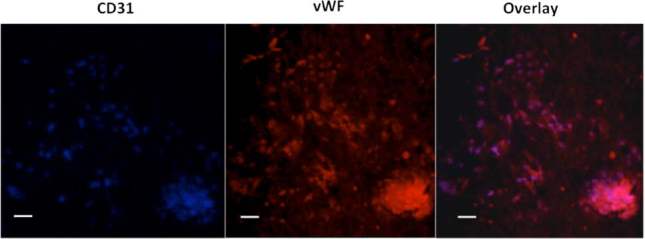
**Culturing HUVECs on POSS-PCU-CD34.** Growth and proliferation of HUVECs were observed on POSS-PCU-CD34 even after being exposed to physiological flow conditions. Scale bar represents 40 μm.

Endothelial progenitor cells were shown to adhere and differentiate on CD34-POSS-PCU polymer films. In contrast, pure POSS-PCU films, and non-specific IgG-POSS-PCU films did not show any EPC capture. Results indicated that only CD34-POSS-PCU were able to capture EPCs, and also to serve as a platform on which growth and proliferation were maintained (Figures 12 and 13). HUVECs were also cultured on CD34-POSS-PCU films, and confocal microscopy revealed that the polymer was able to support its growth and proliferation of these cells (Figure 14). The large agglomeration of cells on POSS-PCU-CD34 panels were possibly due to protein multiplexing, although further evaluation is needed to confirm this.

**Figure 15:**
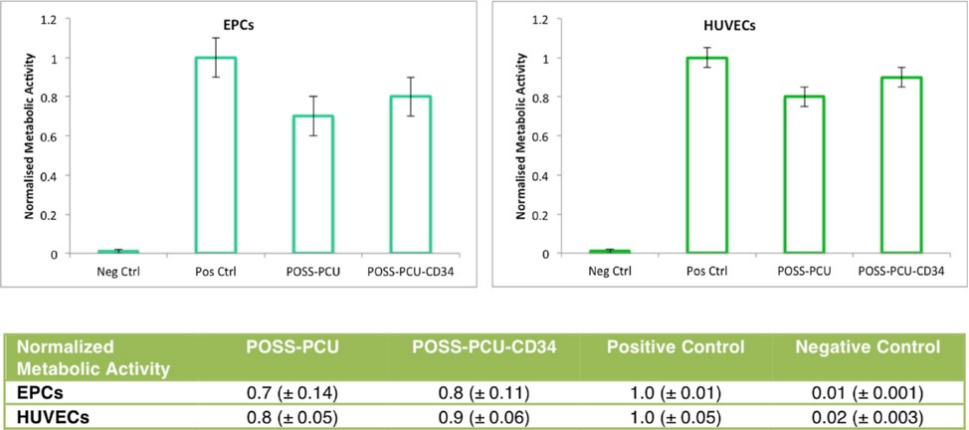
**Assessment of biocompatibility.** alamarBlue showed that EPCs and HUVECs grew and proliferated well on both POSS-PCU and POSS-PCU-CD34 films.

The AlamarBlue® assay was conducted to assess biocompatiblity, and it was shown that cells attached to both POSS-PCU and CD34-POSS-PCU demonstrated a favourable level of normalized metabolic activity, similar to that of positive controls (Figure 15). Cells growing on blank tissue culture plates without polymer films were used as a positive control, while 100% ethanol was used as a negative control. Metabolic activity was normalized to positive controls. Metabolic activity was assessed for both EPCs and HUVECs. Normalized metabolic activity for EPCs on samples were as follows; POSS-PCU: 0.7 (± 1.4), POSS-PCU-CD34: 0.8 (± 0.11), positive control: 1.0 (± 0.01), negative control: 0.01 (±0.001). Normalized metabolic activity for HUVECs were as follows: POSS-PCU: 0.8 (± 0.05), POSS-PCU-CD34: 0.9 (± 0.06), positive control: 1.0 (± 0.05), negative control: 0.02 (± 0.003). Normalized metabolic activities of cells cultured on both POSS-PCU and POSS-PCU-CD34 were statistically significant from controls (p < 0.05).

Therefore, results indicate that both pure POSS-PCU and POSS-PCU-CD34 were able to support the growth and proliferation of EPCs and HUVECs, with a level similar to positive control.

### Stability of antibody attachment in physiological flow conditions

**Figure 16:**
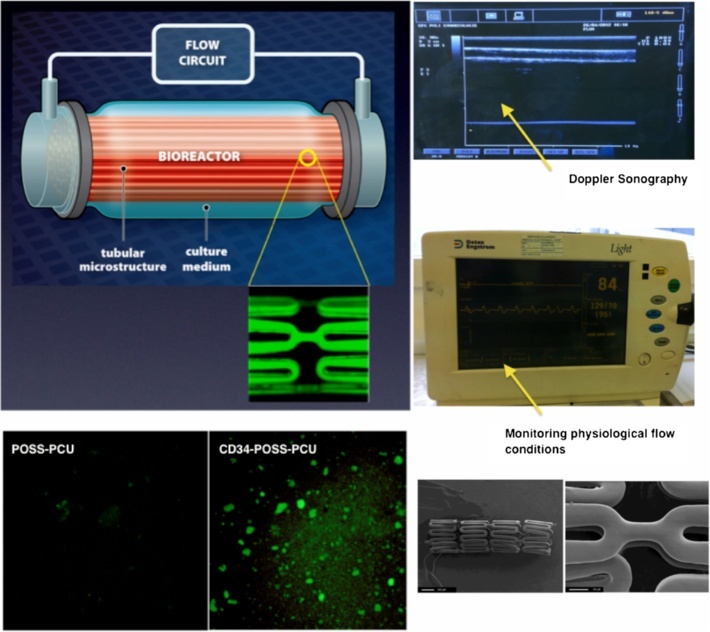
**Stability under physiological flow conditions.** POSS-PCU and CD34-POSS-PCU coated stents were placed in a flow circuit, calibrated to mimic physiological flow conditions, for 28 days. Confocal microscopy using fluorescent QDs on retrieved films showed the presence of anti-CD34 antibodies on the surface even after being exposed to dynamic flow conditions.

To evaluate the robustness and stability of the covalently-linked antibody on POSS-PCU surfaces, antibody-POSS-PCU-coated stents were placed in a custom-built flow circuit to mimic physiological flow conditions in human arteries (Figure 16). Stents were removed after 28 days and confocal microscopy revealed that antibodies were still attached to the surface, confirming the stability even under flow conditions. Preliminary data suggested that it was viable to culture EPCs in the bioreactor subject to flow conditions; however, the levels of EPCs were too low and therefore more optimization of this experimental technique is needed. Nevertheless, we have successfully demonstrated the stability of the immobilized anti-CD34 antibodies under flow conditions.

## Conclusions

The concept of EPC capture for accelerated endothelialization represents a novel method for restoring vessel physiology in the field of regenerative medicine. This study has sought to assess the feasibility of functionalizing a POSS-PCU nanocomposite polymer with an endothelial progenitor cell-specific antibody. We selected anti-CD34 antibodies as we had conducted preliminary work with other EPC-specific antibodies, including anti-CD133 and vWF, and found the capturing efficacy was not as potent as anti-CD34. Furthermore, anti-CD34 antibodies are currently used commercially in an endothelial progenitor cell capturing stent, Genous™ Stent [36]. However, it is important to note that the Genous™ stent is not yet approved by the Food and Drug Administration (FDA), which highlights the fact that more research has to be conducted regarding the actual therapeutic effects of EPC capture technology, compared to conventional DES and BMS [37]. Furthermore, it is also imperative for the polymer coating on stents to be highly biocompatible and haemocompatible. With the recent health scare and public outcry regarding the high failure rates of uncoated metal hip implants [38] and the rupturing of non-medical grade Poly Implant Prosthèse (PIP) breast implants [39], intense research into polymer materials for medical applications are underway [40].

We recognize that a limitation of this study is that it is conducted *in vitro* rather than *in vivo*. Furthermore, it has been previously shown that the levels of circulating EPCs in peripheral blood are extremely low (<0.002%) [41], and therefore one way of increasing the levels of circulating EPCs is via the administration of granulocyte colony-stimulating factor (G-CSF) [42]. However, whether or not this would work in tandem with EPC-capturing stents remains to be seen. Future work would be done with regard to the following areas: anti-CD34 antibody viability in the manufacturing process; stability of the nanocomposite system through various sterilization techniques (e.g. ethylene oxide and gamma irradiation); shelf-life and ageing assessment (e.g. H_2_O2/CoCl_2_ system); mechanical engineering aspects of POSS-PCU to show robust integration and bonding to metal struts without cracks or fractures; *in vivo* biostability of the entire system in an animal model.

An ideal nanocomposite polymer material for coating stents for cardiovascular purposes should be highly biocompatible, haemocompatible, and also non-biodegradable to prevent particles and by-products from leaching into biological systems. We have previously demonstrated POSS-PCU to be suitable for biomedical applications, and ideal for use as various cardiovascular devices such as cardiac valves [43] and vascular grafts [44].

Other studies have proposed the use of antibody-functionalized polymer platforms, such as polyethylene glycol (PEG) [45], polylactic acid (PLA) [46], and collagen [47] for EPC capture. However it is important to note that if polymer platforms were biodegradable and eroded before a confluent layer of endothelium is formed, it would undermine the purpose of having a protective coating on stents in the first place. Furthermore, polymers must be mechanically robust enough to serve as coatings on stents, as these devices are constantly exposed to high fluid shear stress in the cardiovascular system.

Taken together, we have demonstrated a proof-of concept that anti-CD34 antibody-functionalized POSS-PCU nanocomposite polymer can serve as a platform on which to support the growth and proliferation of EPCs with the aim of achieving a confluent layer of endothelium to mimic native vessel physiology.
